# 

**Published:** 1896-07

**Authors:** 


### CONTENTS.

ORIGINAL ARTICLES:

Cryptorchids and their Castration. By R. C. MOORE, V.S. . . . . . . 509

Nutrition. By E. MERILLAT . . . . . . . . . . . . 516

Horse-breeding. By J. A. NESS, D.V.S. . . . . . . . . . . 522

Homeopathy in Veterinary Practice. By A. V. ABBOTT, D.V.S. . . . . . 529

ABSTRACTS FROM FOREIGN JOURNALS:

Thirteen New Cases of Tuberculosis in the Dog—Some Accidents Observed on Horses of Pure English Stock—Malleination and Tuberculination in Point of a Judicial Examination—Statistics on the Frequency of Neoplasms in the Dog—Arecoline, a New Remedy against Acute Founder—Treatment of Colic with Chloride of Barium—Septicæmia Hæmorrhagica . . . . . . 536-549

REPORTS OF CASES:

Intestinal Obstruction in a Colt. By R. P. LYMAN, M.D.V. . . . . . . 550

Paralysis in a Lion. By CECIL FRENCH, D.V.S. . . . . . . . . 551

Nerve-suturing. By W. E. A. WYMAN, V.S. . . . . . . . . . . 552

Urethral Calculus in a Steer . . . . . . . . . . . . . . 553

EDITORIAL:

The Slower Method the Truer One—Adopt this Measure in the Smoky City—Triumphant Massachusetts—Now for the Queen City of the Lakes . . 554-557

CONTROL WORK:

New Jersey—Pennsylvania—Australia—Canada . . . . . . . . 557

LEGISLATION:

Pennsylvania—New York—National—Oregon—District of Columbia . . 558-561

AMONG THE COLLEGES:

Veterinary Department of the University of Pennsylvania—McKillip Veterinary College—The Veterinary Department of the University of California—The College of Agriculture and Mechanical Arts . . . . . . . 561-562

SOCIETY PROCEEDINGS:

Pennsylvania State Board of Veterinary Medical Examiners—Examination Questions, Pennsylvania State Board of Veterinary Medical Examiners—Veterinary Medical Association of New York County—Keystone Veterinary Medical Association—Missouri Valley Veterinary Association—Schuylkill Valley Veterinary Medical Association—The Connecticut Veterinary Medical Society—Massachusetts Veterinary Medical Association—Pennsylvania State Veterinary Medical Association . . . . . . . . . . . . . . 563-571

U. S. V. M. A. . . . . . . . . . . . . . . . . . 572

PERSONAL . . . . . . . . . . . . . . . . . . 575

